# Brazilian guideline for pediatric cycloplegia and mydriasis

**DOI:** 10.5935/0004-2749.20230049

**Published:** 2023

**Authors:** Ian Curi, Simone Akiko Nakayama, Érika Mota Pereira, Luisa Moreira Hopker, Fábio Ejzenbaum, Ronaldo Boaventura Barcellos, Rosane da Cruz Ferreira, Monica Fialho Cronemberger, Craig A Mckeown, Júlia Dutra Rossetto

**Affiliations:** 1 Department of Ophthalmology, Hospital Federal dos Servidores do Estado do Rio de Janeiro, Rio de Janeiro, RJ, Brazil.; 2 Department of Ophthalmology and Visual Science, Escola Paulista de Medicina, Universidade Federal de São Paulo, São Paulo, SP, Brazil.; 3 Department of Ophthalmology, Hospital São Geraldo, Universidade Federal de Minas Gerais, Belo Horizonte, MG, Brazil.; 4 Department of Ophthalmology, Hospital de Olhos do Paraná, Curitiba, PR, Brazil.; 5 Department of Ophthalmology, Santa Casa de Misericórdia de São Paulo, São Paulo, SP, Brazil.; 6 VER Instituto para Prevenção da Cegueira Infantil, Porto Alegre, RS, Brazil.; 7 Pediatric Ophthalmology and Strabismus Service, Bascom Palmer Eye Institute, University of Miami Miller School of Medicine, Miami, FL, USA.; 8 Pediatric Ophthalmology Department, Instituto de Puericultura e Pediatria Martagão Gesteira, Universidade Federal do Rio de Janeiro, Rio de Janeiro, RJ, Brazil.

**Keywords:** Mydriatics, Refraction, ocular, Infant, newborn, Child, diagnostic techniques, ophthalmological, Midríase, Refração ocular, Recém-nascido, Criança, Técnicas de diagnóstico oftalmológico

## Abstract

Cycloplegia is crucial for reliable pediatric ophthalmology examinations. This document provides a re­commendation for pediatric cycloplegia and mydriasis for Brazilian ophthalmologists. This article was developed based on literature reviews; the clinical experience of Brazilian specialists, as obtained through questionnaires; and the consensus of the Expert Committee of the Brazilian Pediatric Ophthalmology Society. According to the best evidence and formulations available in Brazil, this committee recommends the use of one drop of 1% cyclopentolate plus one drop of 1% tropicamide in children older than 6 months and two drops of 1% tropicamide 0-5 minutes apart for those younger than 6 months. Mydriasis may be increased by a single drop of 2.5% phenylephrine. For retinopathy of prematurity screening, the recommendation is 0.5% or 1% tropicamide, administered two or three times, 5 minutes apart, and 2.5% phenylephrine, used preferably once. In all scenarios, we recommend the use of a prior drop of 0.5% proxymetacaine.

## Abstract (pt)

A cicloplegia é crucial para um exame oftalmológico pediátrico acurado. Este documento visa a fornecer uma recomendação para cicloplegia e midríase pediátrica para oftalmologistas brasileiros. Foi desenvolvido com base em revisão literária, na experiência clínica de especialistas brasileiros, por meio de questionários, e no consenso do comitê de especialistas da Sociedade Brasileira de Oftalmologia Pediátrica (SBOP). De acordo com as melhores evidências, este comitê recomenda o uso de uma gota de ciclopentolato 1%, mais uma gota de tropicamida 1% em crianças maiores de 6 meses e duas gotas de tropicamida 1% com intervalo de 0-5 minutos para menores de 6 meses. A midríase pode ser potencializada por uma gota de fenilefrina 2,5%. Para o rastreamento da retinopatia da prematuridade, a recomendação é tropicamida 0,5 ou 1%, duas ou três vezes, com 5 minutos de intervalo, e 2,5% de fenilefrina, preferencialmente uma vez. O uso prévio de proxymetacaína 0,5% é sempre recomendado.

## INTRODUCTION

To perform an appropriate and accurate pediatric eye examination, adequate cycloplegia is essential, as temporary relaxation of the ciliary muscle enables accurate measurement of the refractive status of children. An ideal cycloplegia would be effective, convenient, and safe. Effectiveness would require maximum ciliary muscle paralysis and adequate mydriasis. Convenience would demand a rapid onset of cycloplegic action and a sufficient duration of effect for the examination to be performed but not so prolonged as to cause patient discomfort. Safety would necessitate the absence of side effects. At present, however, no cycloplegic agent meets all of these qualifications. Therefore, there is a need for a protocol that can provide adequate cycloplegia for the pediatric population.

In Brazil, combination cycloplegic and/or mydriatic drops are not commercially available. The only obtainable medications are 1% tropicamide, 1% cyclopentolate, 0.5% and 1% atropine, and 10% phenylephrine. The absence of combined medication formulations at lower concentrations increases the risk of adverse effects. In addition, no standardized national guideline has been established for pediatric cycloplegia. The objective of the present article is to provide a protocol for cycloplegia and mydriasis in children for use by Brazilian ophthalmologists.

## METHODOLOGY

This pediatric cycloplegia/mydriasis guideline was developed based on the medical literature, the clinical experience of Brazilian specialists (as obtained via questionnaires), and the consensus of the Expert Committee of the Brazilian Pediatric Ophthalmology Society (SBOP). PubMed/Medline databases were searched for articles published in peer-reviewed journals written in Portuguese and English using combinations of the following MeSH terms: “cycloplegia,” “cycloplegic,” “mydriasis,” “mydriatic,” “atropine,” “cyclopentolate,” “tropicamide,” “phenylephrine,” “iris color,” “side-effect,” “adverse reaction,” “adverse effect,” “child, preschool,” “infant,” and “pediatric.”

Selected papers were reviewed by the SBOP Expert Committee. Because of the scarcity of well-designed articles on this topic, the documents considered were not restricted to systematic reviews, randomized controlled trials, or observational studies. To classify the level of evidence and the strength of the recommendations, the following method was used: Level I was based on two or more high-quality randomized clinical trials (RCTs). Level II was based on a small number of RCTs; on more than one controlled, but not randomized, study; on more than one RCT of lesser quality; on cohort or case-control studies, preferably from more than one research group or more than one center; or on observations of clear-cut effects in noncontrolled studies. Level III was based on expert opinion, clinical experience, descriptive studies, or cohort or case-control studies of lower quality^(1)^.

A 14-question questionnaire was sent to 337 members of the SBOP and the Brazilian Strabismus Center (CBE), and 136 responses were obtained. Members were asked the following questions about their routines regarding pediatric cycloplegia/mydriasis:

1. What is your subspecialty?
2. Do you use a topical anesthetic before cycloplegic eye drops?
3. At what age do you start using 1% cyclopentolate eye drops?
4. Describe the adverse effects that you have had with cyclopentolate and their frequency.
5. Do you perform different cycloplegic routines for patients with and without strabismus?
6. Do you consider the cycloplegia due to tropicamide equivalent to that obtained with cyclopentolate?
7. Do you consider the cycloplegia due to cyclopentolate equivalent to that obtained with atropine?
8. Do you use atropine in any situation for refraction?
9. Do you use phenylephrine as an adjunct to obtain mydriasis?
10. How long after the cyclopentolate drop do you perform your exam?
11. How long after the tropicamide drop do you perform your exam?
12. Is your routine different for patients with light or dark irises?
13. Do you use cyclopentolate in children with epilepsy? How long after the last crisis?
14. Describe your cycloplegic routine.

Another 10-question questionnaire was sent to a sample of specialists in retinopathy of prematurity (ROP) regarding their routine in obtaining mydriasis in neonatal intensive care units (NICUs).

The results of the literature search and the answers to the questionnaire were critically analyzed by this Expert Committee for the preparation of this guideline. Ethics approval was waived because the study did not require human subject participation.

## RESULTS/RECOMMENDATIONS

### Anesthetic eye drops: are they recommended?

The use of anesthetic drops is intended to reduce the discomfort caused by cycloplegic/mydriatic drops, to improve the child’s experience during the ophthalmological visit, and to enhance the absorption of the drops^(2,3)^. The responses to our questionnaire revealed that 56.6% of the specialists instill anesthetic eye drops before performing cycloplegia. Two double-masked studies with small samples of adults showed a significant reduction in total discomfort scores when both tropicamide (pain scale 5.15 placebo × 2.5 proxymetacaine) and cyclopentolate (pain scale 4.29 placebo × 1.16 proxymetacaine) were instilled after using 0.5% proxy­metacaine (proparacaine) compared with instillation after a placebo^(4,5)^.

Other benefits associated with the use of topical anesthetics include a decreased time to reach the maximum cycloplegic effect^(3)^ and maintenance of the peak of cycloplegia and mydriasis for a longer time.^(6)^ With regard to which anesthetic eye drops are preferable, a randomized, double-masked protocol with 23 adults compared proxymetacaine and tetracaine topical anesthetics. The study showed that the mean pain score was significantly lower with the use of proxymetacaine, albeit with no difference in the cost of medications^(7)^. A survey conducted by the American Association for Pediatric Ophthalmology and Strabismus among its members regarding the choice of the best anesthetic agent for ROP assessments indicated that 63% prefer the use of proxymetacaine, 25% prefer tetracaine, and 3% prefer oxybuprocaine^(8)^. The time of onset and the duration of action of an anesthetic are also relevant for its clinical use. Proxymetacaine has an onset of anesthetic effect of 30 seconds after instillation, and the effect lasts for 15-25 minutes^(9)^.

**Recommendations.** Based on these findings, this Expert Committee recommends the use of one drop of 0.5% proxymetacaine 30 seconds to 1 minute before the instillation of the first mydriatic/cycloplegic eye drop (Level of Recommendation II).

### Atropine, cyclopentolate, or tropicamide. Which is the best choice?

Anticholinergic agents, such as atropine, cyclopentolate, and tropicamide, inhibit the muscarinic actions of acetylcholine in the ciliary muscle (cycloplegia) and the iris sphincter (mydriasis). The human eye has five subtypes of muscarinic receptors (M1 to M5). The ciliary muscle and the iris sphincter each have a complex arrangement composed of all five subtypes, but the M3 type predominates (60-75%)^(10,11)^.

Atropine is a nonselective muscarinic antagonist. By contrast, tropicamide and cyclopentolate probably have different affinities for each receptor subtype, which results in a synergistic effect when used together. The maximum cycloplegic effect occurs within 20-45 minutes with tropicamide, within 30-60 minutes with cyclopentolate, and within 60-180 minutes with atropine. Complete recovery occurs in 6 hours, 24 hours, and 7-12 days, respectively^(11)^.

However, despite the similarity between the effects of tropicamide and cyclopentolate, the window of opportunity for performing the examination is much narrower for tropicamide than for cyclopentolate. One study showed that 2 hours after administration of 1% cyclopentolate, the subjects had regained only 25% of their initial amplitude of accommodation, whereas subjects who had received 1% tropicamide had regained about 70% of their baseline accommodation^(12)^. Thus, although some studies have suggested that cycloplegia is achieved as effectively with tropicamide as with cyclopentolate^(13)^, tropicamide is not recommended for use on its own when refraction is a priority, because its effect is extremely transient.

When used in combination, tropicamide and cyclopentolate allow for a faster cycloplegic onset, a more ample window of peak activity, and a reduced duration of the drug effect in comparison with their use alone. In addition to its greater convenience, the combined formulation is highly effective, as it produces cycloplegia as successfully as cyclopentolate alone and at no additional cost^(14-16)^.

In some countries, 1% atropine remains the gold standard for performing cycloplegia. In Brazil, 41.9% of the specialists recognize that the cycloplegia obtained with 1% cyclopentolate is inferior to that obtained with 1% atropine. Despite this, only 9.6% of pediatric ophthalmologists still use 1% atropine and only in some situations for refraction, as they believe that cyclopentolate is sufficient in most cases.

Atropine is known to have a greater cycloplegic effect than that of other eye drops.^(17,18)^ However, for most routine examinations, this difference might not be clinically significant, and atropine has inconveniently prolonged effects. Thus, cyclopentolate and tropicamide, because of their shorter duration of action, have become more popular options. However, it is important to remember that the use of atropine also leads to a significantly lower mean residual accommodation than is achieved with cyclopentolate and tropicamide combined or with cyclopentolate alone^(15)^. Furthermore, about one-fifth of patients may present a difference of +1.00 diopter or more between the cyclopentolate and atropine refractions in at least one eye^(17)^, and this difference can be meaningful in some clinical scenarios. Conversely, in most cases, the mean spherical equivalent difference is <0.50D^(19)^. Thus, the use of atropine can be reserved for particular circumstances, such as for a suspected residual accommodative component after cyclopentolate and tropicamide cycloplegia in esotropic patients or for accommodation spasms.

There is currently no consensus regarding atropine dosages. A comparison between atropinization administered as two drops (5 minutes apart) in the office (measured 90 minutes afterward) versus 3 days (3 times daily) revealed, on average, values 0.5 diopters higher in the latter group^(20)^. Another study showed no significant difference in cycloplegic refraction between an eight-drop versus a four-drop regimen^(21)^.

In Brazil, Bicas et al conducted a study on the dosage of 1% atropine for cycloplegic refraction. In this study, patients received two drops of 1% atropine, 5 minutes apart, on day zero and used one drop of 1% atropine in each eye, three times a day for the following ten days. Refraction was assessed on days 0, 3, 5, 7, and 10. The study found no significant differences in the refraction measurements. Such findings suggest that no or only low augmentation occurs with cumulative atropine doses^(22,23)^.

Nevertheless, in our questionnaire, 87.50% of the participants responded that tropicamide had an inferior cycloplegic effect as compared with cyclopentolate. This result is in accordance with studies in the literature that showed the superiority of cyclopentolate in obtaining a greater accommodation blockage^(24,25)^.

The American Academy of Ophthalmology (AAO) suggests the use of 1% cyclopentolate only in children older than 6 months and recommends 0.2% cyclopentolate plus 1% phenylephrine in infants younger than 6 months. The AAO states that the required dosage can be higher in heavily pigmented irises and that 1% tropicamide can be used as an adjunct^(26)^. In this context, one important point to highlight is that 78% of Brazilian specialists do not change their protocol according to iris color. One prospective randomized trial compared one, two, and three drops of 1% cyclopentolate and found no statistically significant differences between the treatment groups. The authors concluded that a single drop of 1% cyclopentolate suffices for cycloplegic refraction in children^(27)^. The responses of the Brazilian specialists regarding the age at which they use 1% cyclopentolate indicated that 11.76% use it from birth, 5.88% use it from 3 months onward, 22.06% from 6 months, and 3.68% from 9 months, whereas 44.85% use it only after 12 months.

**Recommendations**. In accordance with the evidence presented, this Expert Committee recommends the use of one drop of cyclopentolate 1% plus one drop of 1% tropicamide to obtain an optimal cycloplegic effect in children older than 6 months (Level of Recommendation II). The use of two drops of 1% tropicamide 0-5 minutes apart is advocated for those younger than 6 months (Level of Recommendation II; Tables 1 and 2). In pediatric cases, 1% atropine can be used as an alternative for cycloplegia in patients with accommodation spasms or with a suspected residual accommodative component not revealed by cyclopentolate plus tropicamide (Level of Recommendation III). The use of 1% atropine twice a day for 3 days seems to be a reasonable dosage to fulfill that goal (Level of Recommendation III).

**Table 1:** SBOP-recommended protocols for infants younger than 6 months

Time	Eye drop
0 min	Proxymetacaine (0.5%)
30 sec-1 min	Tropicamide (1%)
1-6 min	Tropicamide (1%)
30-40 min	Examination

Notes: (i) A single drop of 2.5% phenylephrine should be used to maximize mydriasis when needed (compounded in specialized pharmacies). (ii) The use of a third drop of 1% tropicamide is acceptable if needed.

**Table 2:** SBOP-recommended protocols for children older than 6 months

Time	Eye drop
0 min	Proxymetacaine (0.5%)
30 sec-1 min	Cyclopentolate (1%)
1-6 min	Tropicamide (1%)
30-40 min	Examination

Notes: (i) A single drop of 2.5% phenylephrine (compounded in specialized pharmacies) should be used to maximize mydriasis when needed (ii) The use of a third drop of tropicamide (1%) is acceptable, if needed. (iii) In some specific clinical settings, atropine (1% twice a day for 3 days) can be used according to the decision of the doctor.

### Should we use phenylephrine as an adjuvant to maximize mydriasis?

Phenylephrine is a sympathomimetic agent that acts on alpha-1 adrenergic receptors and has little or no effect on beta-adrenergic receptors. Its mydriatic action occurs due to contraction of the iris dilator muscle, with constriction of the conjunctival arterioles (conjunctival whitening) and activation of the Müller muscle (enlargement of the eyelid fissure) as secondary ocular effects. Its maximum effect is reached after 45-60 minutes of instillation, and the reversal occurs in 6 hours^(28)^. Phenylephrine has no cycloplegic effect; hence, its usefulness is restricted to increasing mydriasis for the evaluation of the extreme retinal periphery (ROP or retinoblastoma) or in cases of poor dilation (uveitis).

Studies have compared different concentrations and combinations of phenylephrine for effective mydriasis and a good safety profile in newborns^(29,30)^. Most guidelines recommend the use of a drop of 2.5% phenylephrine for ROP examination^(31-33)^. However, a systematic review suggests one drop of 1% phenylephrine and 0.2% cyclopentolate as the lowest effective combination regimen^(34)^. Conversely, an RCT that compared three drops of 0.5% tropicamide versus two drops of 0.5% tropicamide plus one drop of 5% phenylephrine reported finding a 1.9 times greater pupil surface area with the latter combination^(35)^.

A prospective randomized study in neonates showed that the use of two drops of 2.5% phenylephrine (at an interval of 5 minutes) is sufficient to significantly increase heart rate and blood pressure. However, when only one drop of 2.5% phenylephrine was used in combination with 0.5% tropicamide and 0.5% cyclopentolate, the mydriasis achieved was sufficient (average of 6.4 mm), and this regimen caused no significant increase in heart rate or systolic pressure in relation to the control group. The treatment group showed only a slight increase in diastolic pressure; this was statistically significant but apparently insignificant from a clinical point of view^(36)^.

Particular attention should be paid to extremely pre­mature infants, extremely low-weight infants, and patients with respiratory distress, as these patients are more susceptible to gastrointestinal side effects. In such patients, vasoconstriction of the blood supply and anticholinergic effect can reduce peristalsis, causing slow gastric emptying, emesis, abdominal distension, and even necrotizing enterocolitis. In older infants and children, the use of one drop of 2.5% phenylephrine, although rarely necessary, presents an adequate safety profile. However, the use of a 10% concentration seems to promote a dangerous increase in the number and severity of side effects, with reports of cardiorespiratory arrest^(37,38)^.

The responses to our survey revealed that 45% of Brazilian pediatric ophthalmologists use phenylephrine as an adjunct to obtain mydriasis under certain circumstances. In Brazil, 2.5% phenylephrine is not commercially available and must be compounded in specialized pharmacies according to current legislation.

**Recommendations.** Based on this evidence, this Expert Committee recommends the use of one drop of adjuvant 2.5% phenylephrine, in addition to the regular protocol, whenever the evaluation of the extreme retinal periphery is necessary or in the presence of poor mydriasis (Level of Recommendation II).

### Premature infant examinations in NICUs

Fifty-nine ROP specialists from different parts of Brazil answered a questionnaire about their dilation routine for premature infants in the NICU. Of these specialists, 35% were pediatric ophthalmologists, 61.4% were retina specialists, and 3.5% were general ophthalmologists. With regard to the use of anesthetic drops, 57.6% declared that they do not use them, whereas 42.4% do use them (27.1% use proxymetacaine, 3.4% use tetracaine, and 11.9% use whichever is available). For cyclopentolate, 94.9% do not use it, whereas 1.7% use 0.2% cyclopentolate, 1.7% use 0.5% cyclopentolate, and 1.7% use 1% cyclopentolate.

Tropicamide is used by most (98%) Brazilian ROP specialists; 59.3% prefer the 0.5% formulation, whereas 37.3% use 1% tropicamide. Phenylephrine is used by 83.1% of those consulted; most (74.6%) advocate the use of the 2.5% concentration, while 5.1% prefer the 1% formulation.

With regard to the number of instillations, 6.8% use each type of drop only once, 44% use it twice, 44% use it three times, and 3.4% use it four times.

Indirect ophthalmoscopy is performed within 20 minutes or less after instillation by 8.4% of the specialists, whereas 27.1% wait for 30 minutes, 42.4% wait for 40 minutes, and 22% wait for 60 minutes.

The questionnaire responses also revealed that, for the drops with concentrations not commercially available, 41.1% of the participants order these from a compounding pharmacy, 39.3% acquire them from their hospital pharmacy, and 16.6% obtain them on their own.

**Recommendations**. For the evaluation of ROP in premature infants with optimal mydriasis, this Expert Committee recommends the use of one drop of 0.5% proxymetacaine, followed by a single drop of 2.5% phenylephrine and two or three drops of 0.5% or 1.0% tropicamide 5 minutes apart (Table 3). The examination should be performed at least 30-40 minutes after the first drop (Level of Recommendation III).

**Table 3:** Mydriasis for premature infants in the neonatal intensive care unit (ROP screening)

Time	Eye drop
0 min	Proxymetacaine (0.5%)
30 sec-1 min	Phenylephrine (2.5%)
6 min	Tropicamide (0.5% or 1%)
11 min	Tropicamide (0.5% or 1%)
30-40 min	Examination

Note: (i) The use of a third drop of tropicamide (0.5%-1%) is acceptable if needed.

### Ideal interval between eye drop instillation and examination

The usual recommendation is to wait 5 minutes between the first and second eye drops to avoid washing out the first drop. This assumption was proven experimentally with albino rabbits, but it should be carefully applied in clinical practice^(39)^.

One randomized study compared two regimens, one with tropicamide plus phenylephrine drops instilled 10 minutes apart and one with concurrent application, and found no statistically significant difference in the pupillary diameter^(40)^. Another clinical study compared the relative pupil surface before and after the administration of one drop of 10% phenylephrine and one drop of 0.5% tropicamide, either immediately or with a 5-minute time interval. The protocol with instillations 5 minutes apart yielded only a 5.6% gain in pupil surface^(41)^.

The timing of the peak action of each medication must be considered when determining the optimum interval between drop instillation and examination under mydriasis. The great majority of Brazilian pediatric ophthalmologists wait at least 30 minutes between cyclopentolate instillation and the examination. Overall, 38.24% wait 30 minutes and 47.79% wait 40 minutes. In the case of tropicamide, 31.62% wait 20 minutes and 44.85% wait 30 minutes.

**Recommendations.** Considering the lack of strong evidence regarding the ideal interval time, this Expert Committee accepts an interval between cyclopentolate and tropicamide drops of 0-5 minutes and an interlude for the examination of 30-40 minutes after the first drop (Level of Recommendation III).

### Side effects and risks of medications

Any medication can cause adverse effects. Therefore, its use should be endorsed when the benefits exceed the risks. However, in addition to the rarity of the occurrence of adverse effects, their severity must be taken into account.

Among SBOP/CBE members, 75% reported mild side effects with the use of 1% cyclopentolate. A large number mention facial flushing, drowsiness, and agitation as sporadic, whereas hallucination is rare. Seven of 136 reported at least one episode of convulsion. When asked about the use of cyclopentolate eye drops in children with a history of epilepsy, 41.18% of doctors contraindicate their use but 58.82% retain their use, with 25.74% using them in any context, 10.29% if seizures have been controlled for 30 days, 3.68% if the control has lasted 60 days, 5.88% if the control has lasted 90 days, and 11.03% if the control has lasted 180 days.

A multicenter survey of German speakers was performed to estimate the likelihood of severe complications (i.e., had to be monitored for several hours) and very severe complications (i.e., caused patients to be admitted to a hospital). A total of 1.7 million cumulative cy­cloplegias over 1112 years of cumulative cycloplegic experience were analyzed. The estimated rates were 1.1:100,000 and 2.7:100.000 for severe psychiatric side effects and 0.5:100,000 and 8.7:100.000 for severe physical side effects using cyclopentolate and atropine, respectively. Therefore, during 30 years of cycloplegic experience with an average of 34 cycloplegias per week, only two severe to very severe complications could be expected with the sole use of cyclopentolate and only 10 with the sole use of atropine. Severe mental complications included intoxication, hallucination, agitation, and depression. Severe physical complications were seizures, asthma, fever, circulatory impairment, and tachycardia. No deaths or permanent damage were reported. However, because of the high diagnostic value of cycloplegic refraction in children, these frequent and short-lasting side effects are viewed as medically acceptable^(42)^.

A Japanese study investigated the incidence rate and side effects of topical 0.25%, 0.5%, or 1% atropine and 1% cyclopentolate for cycloplegia in children aged 15 years or younger. Among 811 patients who received atropine, 8.8% had side effects, most frequently (53.6%) occurring following the initiation of the instillation on the first day. The symptoms included flushing (40.8%), fever (30.0%), and both (15.5%). The risk of adverse effects was higher with the 1% concentration and in the group younger than 1 year. However, no serious reactions were described. Of a total of 2238 patients who received 1% cyclopentolate (one or two instillations), 1.2% had side effects, including drowsiness (37.0%), red eye (14.8%), flushing (11.1%), and redness (11.1%). Hyperactivity, irritable mood, skin sores, and conjunctivitis were also reported. No serious reactions were reported. The reactions were slightly more likely in children younger than 1 year and in patients with systemic diseases, such as Down syndrome^(43)^.

In a Dutch cohort of 3-14-year-old children, 504 patients were administered 1% cyclopentolate + 1% tropicamide (C + T) and 408 had 1% cyclopentolate twice (C + C). Adverse reactions were reported for C + C in 10.3% of the patients and for C + T in 4.8%. Repeated instillation of 1% cyclopentolate, younger age, and low body mass index were associated with higher incidences of side effects, but no serious side effects were reported^(44)^.

Aside from using the proper dosage, studies also recommend the application of pressure to the nasola­crimal sac when adding cycloplegic drops to reduce systemic side effects^(45)^.

**Recommendations**. This Expert Committee does not recommend the use of 1% atropine or 1% cyclopentolate in children younger than 6 months. Although rare, these patients are more vulnerable to severe side effects. For those greater than 6 months of age, the use of 1% atropine or 1% cyclopentolate is safe, except in patients with Down syndrome, neurological problems, history of seizures, or closed-angle glaucoma (Level of Recommendation III).

## DISCUSSION

No protocols currently exist in Brazil for cycloplegia and mydriasis for pediatric eye examination. The purpose of this article is to provide a guideline based on the best scientific evidence, expert consensus, and Brazilian pharmacologic availability.

Cycloplegic refraction is the gold standard for clinical assessment of refractive error in young children^(46)^. There is a statistically significant difference between cycloplegic and noncycloplegic refraction in children, with significantly more hyperopia (less myopia) in the cycloplegic group. This difference is also higher in younger children and in children with greater hyperopia^(47)^. In the pediatric group, up to 18% of all eyes with noncycloplegic myopia become emmetropic after cycloplegia, and 15.7% become hyperopic under cycloplegia^(48)^. Refraction without cycloplegia or with inadequate cycloplegia may alter outcomes in an underplus or overminus direction or can result in an unbalanced prescription between the eyes. This can lead to undesirable consequences, such as amblyopia.

Internationally, formal cycloplegic recommendations can be found only in broad protocols, such as ROP guidelines and strabismus management protocols. For example, the Royal College of Ophthalmologists advo­cates the use of 0.5% proxymetacaine, followed by cyclopentolate (0.5% in children under 6 months and 1% in children older than 6 months) and examination after 30 minutes in pediatric strabismus assessment^(49)^. The College also proposes a mydriatic combination of 2.5% phenylephrine and 0.5% cyclopentolate, instilled as one drop each, in two to three doses, each 5 minutes apart, 1 hour before ROP screening^(33)^.

In Brazil, no combination mydriatic formulations have concentrations suitable for the pediatric public. The available drugs are 1% tropicamide, 1% cyclopentolate, 0.5% and 1% atropine, and 10% phenylephrine. Considering that several of these formulations have the potential for serious adverse effects, especially in infants younger than 6 months, the SBOP recommends the use of only tropicamide in this age group. In children older than 6 months, for efficient cycloplegia, the SBOP recommends the use of one drop of 1% cyclopentolate and one drop of 1% tropicamide. The first instillation should be preceded by one drop of 0.5% proximetacaine, and the examinations should ideally be performed between 30 and 40 minutes of application of the first drop. A single drop of 2.5% phenylephrine should be considered when the retinal periphery assessment is crucial or when dilation is poor. However, the latter medication must be compounded in specialized pharmacies, according to current legislation.

Guidelines are not intended to provide step-by-step medical care or to replace clinical judgment. On the contrary, their intention is to support standards of practice^(50)^. This guideline written by the SBOP should therefore be considered in this context. Adhering to its recommendations will not necessarily produce successful results in all cases. This guideline is also not intended to define or serve as a legal standard for medical care; therefore, it should not be used as a legal resource, as its general nature cannot provide individualized guidance for all patients in all circumstances^(50,51)^.
